# Musical Performance and Biomedical Human Enhancement: Ethnographic Perspectives on Bioethical Questions

**DOI:** 10.1002/hast.70031

**Published:** 2026-05-06

**Authors:** Jennifer Saltzstein

**Keywords:** enhancement, performance anxiety, beta‐blockers, propranolol, musical performance, human values, bioethics

## Abstract

*This article explores the use of biomedical human enhancement in the field of musical performance, focusing on the role of beta‐blocking drugs, which have been used by classical musicians since the 1970s to control the symptoms of performance anxiety, primarily in high‐stakes auditions. Insights gained from in‐depth, structured, ethnographic interviews with professional classical musicians suggest that, unlike professional athletic communities (in which the use of beta‐blocking drugs to control performance anxiety is often banned), classical musicians often view the use of beta‐blocking drugs as a legitimate form of therapy or as a training tool. Although dominant bioethical frameworks for understanding human enhancement focus on values of autonomy, hard work, fairness, and justice, these values were largely absent from the way musicians conceived of beta‐blocker use in their community. At a time when the use of beta‐blocking drugs to control performance anxiety is spreading to job interviews, examining the impact of beta‐blocker use in the field of classical music offers a real‐world case study that shows how cultural values within individual communities shape ethical questions involving biomedical human enhancements*.

## Article

Making music has enhanced the lives of people all over the world, from ancient eras to the present. Music is used nearly universally in worship and celebration. Globally and for centuries, it has been harnessed as a form of therapy to treat illnesses of the body and mind.^1^ The use of music as an enhancement seems to be natural to humans. It may come as a surprise, however, that, in order to perform at their best, many modern professional musicians in the Western art music tradition (what is colloquially called “classical music”) rely on medications that are, in other cultural spheres, classified as biomedical human enhancements. Human enhancement has been defined as “an intervention—a human action of any kind—that improves some capacity (or characteristic) that normal human beings ordinarily have or, more radically, that produces a new one.”^2^ Enhancement allows a person to “reach beyond health to enhance their physical or cognitive capacities, to lift themselves above the norm.”^3^ Preventing disease (even if accomplished by modifying the genes of an embryo) is thus considered therapy rather than enhancement. Further, biomedical enhancement differs from other methods of enhancement: “a *biomedical* enhancement uses biotechnology to cause an improvement of an existing capacity by acting directly on the body (including the brain).”^4^

The biomedical human enhancement that professional classical musicians use most often is the beta‐blocker. The use of this drug within this community is in keeping with the definitions of enhancement put forth by bioethicists. Beta‐blocking drugs act directly on the body to reduce physical symptoms of nervousness, improving a musician's capacity to achieve a peak performance in a situation during which nervousness might otherwise interfere. The use of beta‐blockers as a biomedical human enhancement by Western art musicians has been ongoing since the 1970s. Musicians often experience a fight‐or‐flight response before high‐stakes performances (commonly known as “stage fright” or “music performance anxiety”). This may involve accelerated heart rate, increased respiration, and other symptoms that can interfere with peak musical performance. It is common for professional classical musicians to use propranolol and other beta‐blocking drugs to prevent the physical symptoms of the fight‐or‐flight response. The drug thereby allows a nervous musician to perform as they would in a situation in which they were not nervous.^5^ Examining the impact of decades of beta‐blocker use in the field of classical music offers a real‐world case study demonstrating how biomedical human enhancements can be diffused within a community and reframed according to that community's values.

Surveys suggest that beta‐blockers are now used by a majority of orchestral musicians, particularly in the competitive context of the audition, and that their use has increased dramatically over time.^6^ Listening directly to the views of performing artists illuminates how these communities understand and frame biomedical human enhancements, their motivations for pursuing them (or eschewing them), and how the presence of biomedical enhancements within a community alters incentives and behaviors. Bioethicists have tended to examine human enhancement in ways that are speculative and oriented toward the future.^7^ In contrast, this article focuses on empirical evidence derived from structured, ethnographic interviews with high‐level performing musicians conducted via video calls in 2023 and 2024. The issues of fairness and justice that dominate speculative bioethical literature on human enhancement were largely not viewed as significant by the musicians I interviewed. Most did not conceptualize beta‐blocker use, even in competitive situations, in moral terms. We will see that this is because many musicians frame beta‐blockers as therapies or as tools. Excellent musicians who struggle with performance anxiety have clearly benefited from the use of beta‐blockers, as have audiences who have experienced their artistry. At the same time, the wide diffusion of human enhancements among high‐level classical musicians has created pressure to use them and has compromised values prized within this community such as artistic risk and spontaneity. Since the use of beta‐blocking drugs is spreading to job interviews in other, much larger, fields,^8^ examining professional musicians’ attitudes toward this class of drugs also provides timely insight into ethical and moral questions surrounding the everyday use of biomedical performance enhancements.

## Beta‐Blocker Use by Musicians: Therapy or Enhancement?

Musicians’ use of beta‐blockers sits on a murky spectrum between therapy and enhancement. The most common beta‐blocking drug, propranolol, neither increases a musician's technical ability to play their instrument nor eliminates psychological symptoms such as fear and negative thinking. It can, however, be viewed as enhancing performance because it removes physical barriers to excellent performance such as elevated heart rate, sweating, muscle tremors, and shaking of the breathing column. The drug thereby increases the likelihood that a musician will achieve a peak performance in a situation during which, without the medication, they would ordinarily be less likely to perform at their personal best. It allows the musician to play the way they do in the practice room, even in a high‐stakes, stressful performance situation. Philosophers such as Allen Buchanan have described the use of drugs to control symptoms of stage fright as an example of biomedical human enhancement rather than a treatment for illness. “If you're one of the many musicians who take Adderall [*sic*] to steady your hands,” Buchanan comments, “I'll bet that you use this improvement to tackle more difficult pieces, not to make easy ones even easier. The enhancement serves as a higher platform from which you can exert efforts to attain new excellences.”^9^

We will see that musicians often frame beta‐blocker use not as enhancement but as a medical treatment for performance anxiety. But there are good reasons to dispute the way they conceptualize both performance anxiety and the use of beta‐blockers to treat it. Neither “music performance anxiety” nor general performance anxiety are included in the *Diagnostic and Statistical Manual of Mental Disorders*, or *DSM*.^10^ It was not until the year 2000 that “performance anxiety” was mentioned in the *DSM‐IV‐TR*, where it appeared within a description of social phobia, and the definition stressed that, unless the impairment it caused was significant, performance anxiety should be considered normal.^11^ The severity of performance anxiety varies, but experiencing some amount of nervousness prior to performing is a nearly universal experience among musicians of all ages and abilities, making it difficult to classify as an illness.^12^ Psychologist and psychotherapist Dianna T. Kenny has created a scale that is used to diagnose and treat severe cases of performance anxiety among musicians. Kenny does not recommend beta‐blockers as a treatment for musicians who suffer from severe performance anxiety and notes significant drawbacks to the drug. She recommends that the drug be used only under medical supervision and states that it “should be used as a short‐term adjunct” to support people with severe music performance anxiety. If a musician's symptoms are severe enough to require regular use, she argues, then they need psychological treatment.^13^

Surveys suggest that an audition is by far the most common occasion when professional musicians use beta‐blockers and that many musicians use them only during auditions. In 1987, the International Conference of Symphony and Opera Musicians consulted with medical professionals to develop a survey addressing stage fright and relationships between nervousness and performance generally. Twenty‐seven percent of the musicians who responded reported that they used beta‐blockers to control nervousness. When the organization ran the same survey again in 2015, 70 percent of the musicians who responded reported having tried beta‐blockers, and 90 percent of the musicians who had tried them said they would consider using the drug for auditions. A much smaller percentage (36 percent) listed orchestral performances (noncompetitive but high‐profile situations) as contexts within which they would consider taking the drug.^14^

The widespread use of beta‐blockers in competitive situations among Western art musicians contrasts sharply with its use in sport, where beta‐blocker usage in competition is usually framed as a performance enhancement and is tightly regulated. Beta‐blockers have been banned by the World Anti‐Doping Agency as well as individual sporting organizations ranging from the Professional Golfers’ Association to NASCAR to the World Bridge Federation.^15^ In an interview in 2012, Greg Norman (winner of twenty PGA championships) described the impact of beta‐blocking drugs on his sport prior to their banning: “In my day, lots of guys were on beta‐blockers …. [I]t wasn't openly acknowledged, but it was obvious to the rest of us. A guy's personality would change. In practice rounds or friendly matches, we'd see the real guy under stress. Then in competition, he was like a different, calmer person. Those guys were trying to take the nerves out of the game.”^16^ Addressing the question of how beta‐blockers enhance performance in golf, the PGA anti‐doping manual states, “Athletes may misuse beta‐blockers to decrease heart rate, steady nerves, and stop muscle tremor. Beta‐blockers can decrease anxiety to help control various fine motor skills.”^17^ The organization grants exceptions when the drug is being used to treat hypertension, but the process for obtaining exemption requires a diagnosed medical need. In professional golf and in many other sports, beta‐blockers are viewed unambiguously as performance‐enhancing drugs. In these communities, the use of beta‐blockers as a biomedical human enhancement is commonly viewed as interfering with the integrity of the sport and with the goal of providing a clean, level playing field for athletes.

Yet the same class of beta‐blocking drugs is being used widely in competitive situations in Western art music. Why are attitudes toward beta‐blockers so different among professional musicians? Like golf championships, auditions for major orchestras are also international, high‐stakes competitions between highly skilled participants. As in golf, succeeding in professional musical competitions requires exquisite control of fine motor skills. Whereas professional music organizations have proposed ethical guidelines for orchestral auditions, abiding by them is voluntary, and the guidelines do not address issues of performance enhancement, focusing instead on the transparency of the audition process.^18^ The use of beta‐blocking drugs in competitive musical performance has never, to my knowledge, been banned even though it should arguably raise many of the same ethical questions that it raises in professional sporting competitions. Musicians’ discussions of the use of beta‐blockers in their field make vivid how cultural values within individual communities shape ethical questions involving biomedical human enhancements.

## Ethnographic Insights

The following discussion is based on structured, ethnographic interviews with fifteen professional musicians. Each interview lasted between 45 and 120 minutes.^19^ My interviews revealed a range of attitudes on a variety of topics related to competition and enhancement in Western art music performance. The participants were all professional musicians, and the group included conservatory students, soloists, tenured orchestral musicians, freelance musicians, and university professors of applied music. I selected these musicians based on information about their careers and performing experience found on their professional websites and contacted them individually via email. The musicians lived and worked in different regions of the United States and were diverse in terms of racial and gender identification. All had undergone years or even decades of training on their instruments. None explicitly framed the use of beta‐blockers in competitive musical situations as a form of human enhancement, and few invoked the language of fairness or justice when discussing this class of drugs. On the contrary, most of the professional musicians I interviewed conceived of the drug straightforwardly as medical therapy or supported its use as a form of training technology. Yet other comments musicians made during my interviews suggested that my participants saw an enhancement problem and revealed qualms about using the drug.

I selected participants who played woodwind instruments because this group is among the most competitive of orchestral musicians. Professional orchestras that employ twenty to thirty violinists (or more) rarely hire more than two to four flautists or clarinetists. Yet the employment pipelines for the flute and clarinet are equally large compared, for example, to the string pipeline. Young musicians in the United States who aspire to perform Western art music professionally tend to start their training in the secondary school system, where large wind ensembles and marching bands are common. Instruments such as the clarinet and flute are needed in large numbers in wind ensembles and bands because they perform the same function that violins and violas do within the orchestra—they are used to fill out the ensemble's sound, balancing much louder brass and percussion instruments. The demand for specific instrument types shifts once student musicians decide to seek professional employment opportunities to perform in large ensembles. In the United States, professional wind bands (such as military bands) are fewer in number than orchestras. Many performers also attach greater prestige and artistic value to performing orchestral music. Therefore, the pipeline for woodwind instrumentalists in professional orchestras could be better described as a funnel, in which aspiring professional musicians outnumber available jobs by very large margins. According to the musicians I interviewed, it is not unusual for a single opening for a professional clarinet or flute player in a professional orchestral ensemble or military band to command one hundred fifty to two hundred highly trained applicants invited to audition.

It can be difficult for nonmusicians to understand the level of pressure associated with professional auditions for large musical ensembles. In the first round, a panel of adjudicators may need to cut an initial group of one hundred fifty or more applicants down to the ten musicians who will advance to the next round. In orchestral auditions, applicants are asked to play brief excerpts of the most technically difficult passages from the standard orchestral repertoire. The musicians I interviewed agreed that, to succeed during this first round, performers would need to play with flawless technique, meaning perfectly accurate pitches, rhythms, articulations, tempi, and intonation. Errors in any of these parameters would likely result in failure to advance. In the words of a frequent adjudicator, the first round is focused on “looking for problems” in order to cut the enormous initial field into a smaller group that can be assessed on broader measures of artistry. The applicants would have spent weeks or months preparing for the audition and would have paid for their travel to the audition site. Overall, the musicians I spoke with described the audition situation as maximally stressful. Even among those who had personally obtained a position through this process, most of my interviewees indicated that they wished never to audition again. Musicians describe being selected through an audition for an orchestra or military band as “winning” the job. Their language is apt since, in many ways, the process is as competitive and difficult as winning a professional athletic competition.

The experience of nervousness before significant performances was common among my interview cohort, and for some, it could be significant enough to mar their performance quality. Most assumed that, in an orchestral audition, they would be competing against colleagues who were using some form of medication to eliminate the physical symptoms of nervousness—performers who were “on something.” As one orchestral musician put it, “I think that's almost universal—people show up to auditions with some sort of drug.” Because beta‐blockers are used internally, they are concealed. In a situation in which usage may not be discussed openly, this concealment can increase assumptions about who is or is not using the drug as a biomedical enhancement. For orchestral musicians, the value that the beta‐blocker provides is consistency. For wind players, the body and the instrument are directly connected. By eliminating the shaking of the air column or the fingers, a beta‐blocker has the potential to render a performer's body more consistent and reliable. An orchestral musician who used beta‐blockers in most high‐stakes performing situations (both in auditions and high‐profile solo performances) explained that taking a beta‐blocker “allows me to trust my body in the performance in a way that it was a lot more variable before. So, I wouldn't say that it's necessarily enhancing, but it allows me to do what I know I can do. Whereas before, I might take extra breaths or something … or maybe I'd be conservative on a tempo because I couldn't quite trust my hands. That kind of thing.” I also spoke with a graduate student from a major conservatory who did not use beta‐blockers but relayed to me their conversations with classmates who used them regularly. This student said that beta‐blockers allowed their classmates to “factory out” consistent performances.^20^

Overall, the attitudes my interviewees expressed toward colleagues who used beta‐blockers (even in the competitive arena of the audition) were marked by acceptance, compassion, and an assumption that the use of the drug was therapeutic. As one musician said, “If it [a beta‐blocker] works for you, use it. Absolutely. Uh. It doesn't make you a better musician … . It doesn't make you have better technique. It's not some miracle performance pill. I don't see the issue in it. If it is able to knock a couple degrees off your nerves and it is going to allow you to perform better, then you *should* use it.” Something like this view was held by most of my interviewees, although the rationales behind it differed. Some performers communicated their belief that beta‐blockers were harmless and legal. (The belief that they are harmless is, however, unfounded. Side effects and counterindications exist for propranolol even in low doses.^21^)

Others conceptualized the drug as a fully legitimate form of therapy. One musician explained, “I think for a professional musician who, presumably, is an adult, and is capable of making their own informed decisions, and they consulted, you know, with their doctor, medical team, you know, and they understand the risks and side effects, then it's just like any other treatment to my mind.” Another performer compared a hypothetical musician's reliance on beta‐blockers to her own use of an inhaler as an asthma sufferer, conceptualizing the drug straightforwardly as a form of medical treatment and implicitly comparing musical performance anxiety to the life‐threatening condition of asthma. Others conceptualized the drug as a type of musical equipment. Some clarinet, saxophone, and bassoon players attach their instruments to neck straps or other harnesses to redistribute the weight of the instrument, relieving pressure from their hands while they play. One of my interviewees compared using beta‐blockers to using neck straps, arguing that a beta‐blocker was like any other piece of equipment that enables a musician to perform at their best. These views suggest the normalization of beta‐blockers within the musical community and, often, the rejection of the kinds of moral questions that surround their use as performance enhancements in sport.

Although none of my interviewees described beta‐blockers as performance enhancements or objected to the use of the drugs by peers with whom they were in direct competition, some of their comments give good reason for us to frame beta‐blocker use as an enhancement in Western art music. Several compared the use of beta‐blockers by musicians directly to other forms of human enhancement, such as drinking coffee and receiving Botox injections. One compared using beta‐blockers to a technological realm of musical human enhancement—the recording studio. He explained that using beta‐blockers was akin to adding reverberation to a recording to enhance a musician's sound quality.

Indeed, the outward tenor of acceptance among my interviewees belied deeper ambivalence about the use of the drug in competitive situations, suggesting a latent view that the drug was, in fact, functioning as an enhancement. One freelance orchestral musician I interviewed had used beta‐blockers regularly in a variety of performance situations. This musician was quick to tell me, however, that they had won a national audition before ever using the drug. They seemed to want me to know that they could win clean. I also interviewed a tenured woodwind player in a regional orchestra who conveyed their perception that most musicians would not want their colleagues to know that they had won an audition while using beta‐blockers. This musician suggested that knowledge that a beta‐blocker had been used might diminish the accomplishment in the eyes of one's peers: “I think if it's somebody you're just colleagues with, you're not really going to talk about it [using beta‐blockers]. *Especially* in an audition. Yeah. [*Long pause.*] Because nobody ever wants to feel like they won a job because, um, you know, because they were taking a beta‐blocker. Which is not … [*pause*]. There's no way to make this illegal because it's not … [*pause*] it's not really a sport. This isn't the Tour de France. Right?” This comment suggests that performers who use beta‐blockers in auditions still want their peers to believe that they won the job “clean.” It suggests that there is an understanding within the community that the drug does enhance performance, and it implies latent ethical misgivings or concerns about honor.

My interviews also demonstrated that, within this community, there is reluctance to speak openly about the drug and its role. Some musicians stressed that they discuss their use of beta‐blockers only with close colleagues or that they do not discuss the drug with colleagues who play the same instrument they do. Indeed, I often heard ambivalence about discussing beta‐blockers with students (even when the students were adults). Several performers said that they did not discuss the drug with their students, and some did not believe that other music teachers should discuss beta‐blockers with students. One musician and teacher said, “I don't think that most people talk to their students about it. Or don't recommend it to their students. And so that's kind of curious, considering that most professionals … or at least a good percentage of professionals I know do use them. So, I don't know. I guess there is some sort of negative feelings about it.” I interviewed musicians who used beta‐blockers routinely and were open about this with their peers but who said they did not discuss the drug with their students. In some cases, this reluctance stemmed from their view that the bond between music teachers and their students can be profound and that music teachers often have enormous influence over their protégées. A professional musician and private music teacher explained, “I think it can be a little tricky if there are younger students whose very influential mentors are suggesting this, you know, and, um, if they feel uncomfortable, but they also feel like they have to do what their mentor asks them to do.” Other interviewees said that they didn't volunteer information about beta‐blockers but that they would answer questions from their students if they asked directly about the drug.

During the interviews, none of the interviewees expressed the view that using beta‐blockers at an audition was an unfair enhancement. But several said that, when they were younger, they believed that they would be at a competitive disadvantage if they did not use the drug for auditions. A woodwind professor at a conservatory known for preparing students for orchestral auditions told me that students frequently asked about beta‐blockers, believing that the drug would give them a competitive edge in an audition. Students would express their frustration with the audition situation, saying that they didn't want to be the only one at the audition who was performing while nervous. Despite the tendency for musicians to frame the drug therapeutically, comments like these suggest an underlying understanding of its role as an enhancement. Some musicians clearly feel that success in auditions will depend on using beta‐blockers, but they may also believe that, if they were to “win” an audition using a beta‐blocker, their success might be viewed as undeserved.

## Implications for Existing Bioethical Frameworks

Philosophical literature on biomedical human enhancement is marked by considerable enthusiasm for enhancements and has tended to focus on future‐oriented, hypothetical examples. My interviews illuminate a context in which biomedical human enhancement has been in use for decades and has become routine and even expected. In some contexts, the impact of the drug has arguably been coercive, meaning that some musicians feel that, if they choose not to use it, they will be unable to compete with musicians who do. I believe that the historical trajectory of beta‐blocker usage by professional musicians illustrates unanticipated risks attendant on the widespread adoption of biomedical human enhancements—it is a case in which losses to the community may outweigh the gains that enhancement offers to individuals.

Objections to biomedical human enhancement often cite fairness as a concern. In Western art music performance, beta‐blocking drugs are primarily used in competition. As in many competitive human endeavors, questions of fairness in this arena are important to all involved but are not straightforward. Proponents of biomedical human enhancement often note that unearned advantage is everywhere, whether we consider the impacts of socioeconomic inequality or genetic advantages. Although fairness and equality are deeply valued in sport, they are always impossible to realize fully, given individuals’ differing genetic endowments.^22^ This is also true of the competitive world of Western art music performance, where no one would advocate leveling the playing field in an audition or concerto competition by handicapping a musician with perfect pitch. Although dramatic differences exist in the cost of musical instruments and their precision, musicians would find it unthinkable for adjudicators to specify the equipment they must use in an audition. Performers have highly personal relationships with their musical instruments. They spend considerable energy customizing them, and some even think of their equipment as an extension of their hands.

Fairness claims against human enhancement sometimes characterize enhancements as personal goods and as zero sum.^23^ The case of the talented musician who is able to share their gifts with the world with the help of beta‐blockers, however, represents an enhancement that is arguably positive sum, yielding benefits for the nonenhanced (in this case, for audiences). The audition system was not neutral prior to the introduction of beta‐blockers—it gave an advantage to musicians who naturally experienced less nervousness in stressful situations. There are musicians who are capable of rendering performances of the skill and quality that professional ensembles seek in their employees but who cannot consistently deliver such performances during auditions. Beta‐blockers arguably allow talented musicians whose nervousness would otherwise impede performance to fulfill their professional goals, advance the musical art form, and delight audiences, a positive‐sum outcome.

In the arena of the audition, however, the use of beta‐blockers is, my study suggests, better characterized as zero sum. My conversations with musicians suggest that this drug has so saturated the culture of the audition that its use has become expected, or, as one of my interviewees described it, “near universal.” The nonenhanced musician can reasonably conclude that the enhanced musicians against whom they are competing will be considerably more likely to achieve a peak performance. Meanwhile, which auditioners are using a beta‐blocker will not be apparent to judges or other competitors—unlike a neck strap, the use of beta‐blockers is concealed. This means that a musician's sense of freedom to perform without the enhancement is limited by the mere knowledge (or belief) that others are using the drug. The bioethical questions that arise from beta‐blocker use in music are therefore akin to those surrounding the use of performance‐enhancing drugs in sport, which Thomas H. Murray has argued are inherently coercive: “when some choose to do what gives them a competitive edge, others will be pressed to do likewise, or resign themselves to either accepting a competitive disadvantage or leaving the endeavor entirely.”^24^

Other bioethical frameworks maintain that safe, legal regimes in which biomedical enhancements could be administered to the public—what Buchanan calls the “Enhancement Enterprise”—could eliminate most ethical concerns. Many biomedical enhancements emerge when physicians and patients notice beneficial side effects of drugs created to treat disease. Off‐label prescription of such drugs for purposes of human enhancement creates a back door through which the drugs enter society. Rather than implicitly enabling the back‐door use of enhancement, it would be preferable, Buchanan argues, for society to create a “front door” through which biomedical enhancements would be tested for safety, regulated, and made widely available for general societal improvement while reducing the safety concerns and the inequitable distribution of back‐door enhancements accomplished through off‐label prescription.^25^ The use of beta‐blockers by musicians constitutes a classic case of an enhancement entering society through the back door. The beta‐blocking drug propranolol was introduced to treat angina pectoris and later used to treat cardiac arrhythmias and hypertension, as well as to prevent sudden death after myocardial infarction. Later, migraine headaches were added to the list of on‐label conditions it could be used to treat, and finally, symptoms of stress‐induced anxiety were, too.^26^

Based on the insights from my interviews, the creation of a front door for beta‐blocker usage by musicians would have virtually no impact on the role that biomedical human enhancements are playing in professional auditions. Musicians are most likely to take beta‐blockers before an audition, which is, by most accounts, the most high‐stakes, stressful, competitive situation they face. This means that, even if a front door were in place, musicians could still reasonably assume that all their competitors in an audition had obtained the enhancement and were “on something.” Musicians would probably also conclude that, if they did not also take the drug, then they would be at a competitive disadvantage. The situation for musicians thus mirrors conditions that Murray described as coercive when they occurred in the arena of sport. The creation of a front door might provide peace of mind regarding the long‐term safety of the drug among the musical population, but it would do nothing to eliminate the ethical concerns noted earlier about coercion. Writing about beta‐blocker use by musicians in this journal in 1992, Jacquelyn Slomka noted that, at least some of the time, propranolol was being used by musicians to treat anxieties that should be viewed as normal and associated generally with performance. She marked this as cause for concern, arguing, “The creation (by both medicine and society) of an entity called ‘performance anxiety’ that musicians experience and that physicians treat with drugs is what some psychiatrists and anthropologists might call a ‘culture‐bound syndrome,’” meaning the classification as an illness of a cluster of symptoms that would not be viewed as an illness outside a particular cultural context.^27^ Moreover, Slomka noted that there is a coercive potential in such a situation, much as with performance‐enhancing drugs in athletics. She voiced concern about a situation wherein one musician's use compels another's and the whole community is compelled to use the drug “to return to the state of equilibrium that existed before drug use.”^28^

As I noted, in the decades since Slomka voiced these concerns, surveys have shown that the use of this drug has increased dramatically in the population of professional classical music performers. Further, my interviews suggest that coercion plays a role in the drug's continued use within the community. This is problematic because most of the musicians I spoke with who had used beta‐blockers said that they did not enjoy playing on them. Although nearly all my interviewees experienced nervousness about auditions and other high‐stakes performances, some chose not to use beta‐blockers or had chosen to discontinue their use. Their reasons varied. Some explanations foregrounded honor. One freelance musician said they were determined “not to go the drug route” because older generations of performers succeeded without the help of drugs. Another explained that he felt connected with a historical tradition of music making, noting that the orchestral musicians of previous generations whom he admired had succeeded without pharmacological interventions and he wanted to do the same. Others explained they felt the experience of performing while on the drug was less enjoyable and less edifying. Some expressed that playing on beta‐blockers made them feel flat and unfocused or said that they missed the adrenaline they associated with performing. A professional orchestral musician who had once used beta‐blockers regularly in auditions and even rehearsals told me that they now find them “mind‐numbing and, um, stupid making. They pull things down so you just, you feel flat. But you're not feeling nervous. But you still *are* nervous. Your mind is still … . [Y]ou're just not present.” Despite qualms like these, there is a continuing perception in this community that beta‐blockers are being used widely, fueling more interest in them and more use of them. These comments serve as a reminder that an enhancement does not necessarily make one better off overall.

Both proponents and critics of biomedical human enhancement argue that we should exercise caution when enhancement endangers valued social practices. Values in danger in this case, according to the musicians I interviewed, include pleasure in performance as well as artistic spontaneity and risk—all of which can be diminished by beta‐blocker use. Musical notation provides precise information about the pitches, rhythms, and tempi (relative speed) that a performer should play. Musicians describe accurate execution of these musical parameters, alongside other fundamentals, as “technique.” Many other elements of an artistic rendering remain in the realm of the performer's interpretation. A “riskier” performance may push accepted interpretive boundaries in ways that are artistically invigorating but may risk flawless technical execution. According to one interviewee, “Imperfections show that a performer took artistic risks.” As another put it, “There is beauty in failure.” In Western classical music, artistic risk is endangered by many factors, not only beta‐blocker usage. For example, because of the ubiquitousness of recording technology, many musicians assume they are always being recorded. This creates increasing pressure to play with flawless technique at all times, a condition not particularly conducive to artistic risk taking. Beta‐blockers are also a contributing factor. By rendering the performances of those who use them more uniform and reliable, the widespread use of beta‐blockers helps—as edited recordings also do—to train the ear for technical perfection. This increases expectations of technical consistency on the part of musicians and listeners, a development that at least some of my interviewees worry is inconducive to artistic risk.

The values that the musicians I interviewed attached to beta‐blocker usage in their community diverged notably from those that have been the focus of bioethical literature on human enhancement. Unlike bioethicists (and unlike athletic organizations), few musicians I spoke with viewed a beta‐blocker as an enhancement; most conceptualized it as a form of equipment or medical treatment. Concerns with fairness, justice, and what constitutes a level playing field were never the focus of their remarks. Values around autonomy and hard work were not viewed as relevant, since the drug was not perceived as eliminating the need for practicing.^29^ Even those I spoke with who had decided to stop using the drug or were critical of its impact in their field did not censure other musicians who were using it (even in high‐stakes, competitive situations). The most common moral value they expressed was solidarity. I spoke with unenhanced musicians who felt strong solidarity with their enhanced peers, and vice versa.^30^ Performers expressed resigned acceptance of the rules of the game they played, compassion for colleagues, and a nonjudgmental sense that everyone was simply doing their best to thrive in a difficult situation. Moral values that loom large in bioethical literature were thus virtually absent or not viewed as important by those directly involved.

## Practical Implications and Recommendations

How can it be that a golfer who uses beta‐blockers to eliminate hand tremors before a tournament is abusing a performance‐enhancing drug and will be disqualified from competition while a musician who uses the same drug for the same purpose before an orchestral audition is accessing legitimate medical therapy? Both people are involved in high‐stakes competitions. For each, success will ultimately depend not only on talent and thousands of hours of preparation but also skill at performance itself—the ability to muster one's personal best in a stressful situation. Musicians use the same drug in the same way that athletes do, in pursuit of similar goals and in response to the same physical sensations. The use of beta‐blocking drugs in the musical profession presents us with a puzzle. I will close by arguing that the role of the drug in this community is not serving the values of competition or the recommended therapeutic standards.

What if we were to accept the perspective expressed by many of the musicians I interviewed? Beta‐blocker use would be regarded as a legitimate therapy for performance anxiety, not a performance enhancement. Further, musicians who do not use the drug (even those who actively oppose its use) would not have a reason to hold animosity toward colleagues who use it. Their feelings of solidarity may stem from compassion toward colleagues who suffer from what they view as an illness. Questions of autonomy and fairness may seem irrelevant since those who suffer from an illness are not viewed as responsible for it. The lack of openness in discussing the drug among musicians who are in direct competition with one another may reflect stigmas related to mental illness that are attached to the use of the drug.

There are major problems with this view, however. As I discussed earlier, music performance anxiety is not an illness recognized in the *DSM*, and descriptions of general performance anxiety in the *DSM* stress that it should be viewed as normal, not pathological. Moreover, clinicians who treat music performance anxiety as an illness do not recommend regular beta‐blocker use as an effective therapy. Even in severe cases, Kenny (creator of a music performance anxiety diagnostic scale) does not recommend regular beta‐blocker use. She stresses that beta‐blockers should be used only under medical supervision and argues that, if performers have symptoms severe enough to require regular use of the drug, they require psychological treatment. She argues that cognitive behavioral therapy (CBT) is the most effective, lasting treatment for music performance anxiety. Thus, although musicians frame beta‐blockers as therapeutic, surveys (as well as my own interviews) show a very different pattern of use than what medical professionals recommend. Most musicians obtain beta‐blockers from their general practitioner, many use them regularly, and some share their prescriptions with friends.

What if we draw a different conclusion—that the use of beta‐blockers among many musicians in competition is not therapeutic but, rather, an enhancement? It could be the case that the differing framing around beta‐blocker use in the fields of athletics and musical performance is not substantive but instead a matter of cultural framing and enforcement. Comments by my interviewees suggest that, despite explicitly expressed views that the drug is therapeutic and acceptable, there are underlying suspicions that it is also being used as an enhancement and qualms surrounding its role in the field. This is clear in the comments of the orchestral musician who emphasized to me that, although they used beta‐blockers regularly now, they had won an audition without the drug earlier in their career (meaning they could “win clean”). It also manifests in the comments of the orchestral performer who said that musicians would not want their peers to know that they had won an audition while using a beta‐blocker. Such comments might not reflect stigma around the therapeutic use of the drug but, rather, concerns that the “win” would be seen as facilitated by the enhancement and thereby somehow less honorable or deserved.

In addition to raising fairness concerns, these comments resonate with Michael Sandel's observation, in the context of sport, that, “[a]s the role of the enhancement increases, our admiration for the achievement fades.”^31^ The stakes of auditions (which can result in lifetime employment at high pay) are much higher than those of an individual sporting competition. My interviewees from the orchestral field suggest that one reason beta‐blockers are not framed as performance enhancements in classical music is the lack of an entity or mechanism to enforce limits on their use. To repeat the words of one interviewee, “There's no way to make this illegal because it's not … [*pause*] it's not really a sport. This isn't the Tour de France. Right?” The implication is that the use of beta‐blockers by musicians is indeed similar to athletic use of the drug as a performance enhancement, but that, unlike high‐stakes sporting competitions, the orchestral world has no equivalent to the World Anti‐Doping Agency.

I believe that bioethics scholars rightly cite beta‐blocker use by musicians as an example of human enhancement. But regardless of which explanation we find more compelling, the current situation of beta‐blocker use by musicians does not seem to serve the values of either therapy or fair competition.

Professional classical musicians represent a very small portion of the labor force; yet their perspectives demonstrate how the integration of a biomedical human enhancement like beta‐blockers into a field or area can, over the course of just a few decades, become expected. Beta‐blockers are now being used by some job applicants to keep a cool head and project calm in interviews for highly paid positions.^32^ It would be unfortunate if job interviews in other professions were to follow the path of musical auditions, with job seekers assuming that other interviewees were using biomedical enhancements to ensure they could perform at the peak of their abilities. Opponents of the widespread adoption of human enhancements sometimes worry that the enhancement will become a substitute for effort, causing skills to decline. In the case of beta‐blockers, what is enhanced is not musical technique as such, since beta‐blockers do not reduce the need for musicians to hone their technique. The skill that has the potential to decline due to the enhancement is the ability to perform under pressure. This skill is often essential to a professional activity, whether the applicant is a professional musician or an investment banker. If applicants use beta‐blockers to attain a stressful position, it seems likely that their need for enhancement drugs will extend beyond the interview to the day‐to‐day performance of the job.^33^

Sandel argues, “Rather than employ our new genetic powers to straighten ‘the crooked timber of humanity,’ we should do what we can to create social and political arrangements more hospitable to the gifts and limitations of imperfect human beings.”^34^ In the context of Western art music, many changes could make musical culture more hospitable for advanced musical performers. Arts organizations could open more conversations about the role of beta‐blockers in their field and establish norms around their use. Instructors of applied music could teach their students to engage in more frequent, low‐stakes performances so that they are less nervous in high‐stakes musical situations (and thereby less likely to seek medical intervention). Music schools could train musicians for large ensemble performance in numbers that more closely reflect the employment needs of professional orchestras, alleviating the bottleneck in the career pipeline. The performers with whom I spoke are actively working toward these and other positive changes. Those of us who value listening to classical music can help musicians by donating to musical organizations and by advocating for more public funding of the fine arts, thereby creating opportunities for more performers to earn a living by sharing their artistry with audiences.

Medical professionals also have a role to play in this situation. When treating patients for musical performance anxiety, doctors could recommend behavioral interventions such as CBT instead of prescribing beta‐blockers off‐label. Existing clinical research suggests that such therapy is as effective as beta‐blockers in reducing performance anxiety. Unlike beta‐blockers, CBT has no side effects, and its positive impact lasts much longer than the drug.^35^ Although it may be more expensive and time consuming, potentially raising equity issues, it is safer and more effective. Indeed, existing research recommends beta‐blockers for only occasional use, and some psychologists have argued that beta‐blockers actually hinder the effectiveness of CBT by interfering with the patient's exposure to the stressful situation.^36^ Sharing beta‐blockers and using them unsupervised continues within communities of Western art musicians. If general practitioners were to prescribe CBT instead of beta‐blockers for performance anxiety, they would ensure that their patents received a longer‐lasting, more effective treatment while also reducing the supply of beta‐blocking drugs among the musical population.

## Acknowledgments

This article grew from extended dialogue with and bibliographic recommendations from Brian A. Chance, who also read several drafts and provided valuable feedback. I am especially grateful to the busy performing artists who gave generously of their time to be interviewed.

## References

[1] P. Horden , ed., Music as Medicine: The History of Music Therapy since Antiquity (New York: Routledge, 2016).

[2] A. Buchanan , Better Than Human: The Promise and Perils of Biomedical Enhancement (Oxford: Oxford University Press, 2011), 5.

[3] M. J. Sandel , The Case against Perfection: Ethics in the Age of Genetic Engineering (Cambridge, MA: Harvard University Press, 2007), 8.

[4] Buchanan , Better Than Human, 5.

[5] J. Slomka , “Playing with Propranolol,” Hastings Center Report 22, no. 4 (1992): 13–17, at 13. 1506178

[6] Compare the results of the International Conference of Symphony and Opera Musicians survey from 1987 and 2015. J. Beder, “The 2015 Musicians’ Health Survey Results,” Senza Sordino, June 2017, https://www.icsom.org/senzasordino/2017/06/the-2015-musicians-health-survey-results/. See also the review of literature in D. T. Kenny , “Treatment,” chap. 7 in The Psychology of Music Performance Anxiety (Oxford: Oxford University Press, 2011).

[7] This thread runs through many of the chapters in A. Akabayashi , ed., The Future of Bioethics: International Dialogues (Oxford: Oxford University Press, 2014).

[8] C. Caron , “What Doctors Want You to Know about Beta Blockers for Anxiety,” New York Times, April 3, 2024.

[9] Buchanan , Better Than Human, 160.

[10] Kenny has defined music performance anxiety, discussed its relationship to other anxiety disorders, and described the efficacy of a wide range of treatments. See Kenny, “Defining Music Performance Anxiety,” chap. 4 in *The Psychology of Music Performance Anxiety*. Based on decades of clinical experience, Kenny also developed a tool for diagnosing music performance anxiety (the Kenny Music Performance Anxiety Inventory, or K-MPAI). See D. T. Kenny , “The Kenny Music Performance Anxiety Inventory (K-MPAI): Scale Construction, Cross-Cultural Validation, Theoretical Underpinnings, and Diagnostic and Therapeutic Utility,” Frontiers in Psychology 14 (2023): 1–12. 10.3389/fpsyg.2023.1143359 PMC10262052 37325731

[11] See the discussion in Kenny , Psychology of Music Performance Anxiety, 49.

[12] Slomka wrestled with the difficulty in establishing what level of feelings of discomfort and distress surrounding performance was “normal” and what level was a pathologic phenomenon in need of medical treatment. Slomka , “Playing with Propranolol,” 15.

[13] See Kenny , “Treatment.”

[14] Beder , “The 2015 Musicians’ Health Survey Results.”

[15] The World Anti-Doping Agency prohibits the use of beta-blockers in competition in archery, automobile sports, billiards, darts, golf, miniature golf, shooting, skiing and snowboarding, and underwater sports. In the sports of archery and shooting, beta-blockers are prohibited even outside competition in training situations. See World Anti-Doping Agency , The World Anti-Doping Code International Standard Prohibited List (World Anti-Doping Agency, 2024), https://www.wada-ama.org/sites/default/files/2023-09/2024list_en_final_22_september_2023.pdf.

[16] B. Pennington , “Heart Medications May Also Calm Nerves, Keeping Them Banned,” New York Times, November 14, 2012.

[17] PGA Tour , Anti-Doping Program Manual (PGA Tour, 2022), https://qualifying.pgatourhq.com/static-assets/uploads/PCA-763451-2023-PGA-TOUR-Anti-Doping-Manual-Updates-FINAL.pdf

[18] See, for example, the International Conference of Symphony Orchestra Musicians , Audition Code of Ethics (International Conference of Symphony Orchestra Musicians, 1984), https://www.icsom.org/history/docs/Audition_Code_of_Ethics.pdf.

[19] I transcribed all interviews by hand.

[20] Their comments reveal the kinds of attitudes and motivations that bioethicists have supposed drive the demand for biomedical enhancement—the desire to remove certain kinds of uncertainty. Sandel , The Case against Perfection, 3–4.

[21] See the detailed discussion in Kenny , “Treatment.”

[22] N. Frost , “Banning Drugs in Sports: A Skeptical View,” Hastings Center Report 16, no. 4 (1986): 5–10. 3744803

[23] Buchanan , Better Than Human, 159.

[24] T. H. Murray , “The Coercive Power of Drugs in Sports,” Hastings Center Report 13, no. 4 (1983): 24–30, at 27; see also E. Chang, “Why Athletic Doping Should Be Banned,” *Journal of Applied Philosophy* 29, no. 1 (2012): 33-49, at 27. 6629745

[25] Buchanan , Beyond Humanity?, 16–20 and passim.

[26] Slomka , “Playing with Propranolol,” 13. 1506178

[27] Ibid., 16.

[28] Ibid., 15.

[29] My empirical evidence thus does not support the salience of values of hard work and autonomy stressed in Slomka, “Playing with Propranolol,” 14.

[30] The situation does not support arguments that human enhancement harms solidarity. See Sandel , The Case against Perfection, 86–90.

[31] Ibid., 25.

[32] Caron , “Beta Blockers for Anxiety.”

[33] The situation may follow the trajectory of the abuse of ADHD drugs among investment bankers. See A. Saeedy , “The Drugs Young Bankers Use to Get through the Day—and Night,” Wall Street Journal, December 14, 2024.

[34] Sandel , The Case against Perfection, 97.

[35] See A. Ortiz Brugués , “Music Performance Anxiety—Part 2: A Review of Treatment Options,” Medical Problems of Performing Artists 26, no. 3 (2011): 164–71, and Kenny, “Treatment.” 21987072

[36] Kenny , “Treatment.”

